# Dissection of major depressive disorder using polygenic risk scores for schizophrenia in two independent cohorts

**DOI:** 10.1038/tp.2016.207

**Published:** 2016-11-01

**Authors:** H C Whalley, M J Adams, L S Hall, T-K Clarke, A M Fernandez-Pujals, J Gibson, E Wigmore, J Hafferty, S P Hagenaars, G Davies, A Campbell, C Hayward, S M Lawrie, D J Porteous, I J Deary, A M McIntosh

**Affiliations:** 1 Division of Psychiatry, Royal Edinburgh Hospital, University of Edinburgh, Edinburgh, UK; 2 Centre for Cognitive Ageing and Cognitive Epidemiology, University of Edinburgh, Edinburgh, UK; 3 Department of Psychology, University of Edinburgh, Edinburgh, UK; 4 Centre for Genetics and Molecular Medicine, Institute of Genetics and Molecular Medicine, Western General Hospital, University of Edinburgh, Edinburgh, UK; 5 MRC Human Genetics Unit, Institute of Genetics and Molecular Medicine, University of Edinburgh, Edinburgh, UK

## Abstract

Major depressive disorder (MDD) is known for its substantial clinical and suspected causal heterogeneity. It is characterized by low mood, psychomotor slowing and increased levels of the personality trait neuroticism; factors also associated with schizophrenia (SCZ). It is possible that some cases of MDD may have a substantial genetic loading for SCZ. The presence of SCZ-like MDD subgroups would be indicated by an interaction between MDD status and polygenic risk of SCZ on cognitive, personality and mood measures. Here, we hypothesized that higher SCZ polygenic risk would define larger MDD case–control differences in cognitive ability, and smaller differences in distress and neuroticism. Polygenic risk scores (PRSs) for SCZ and their association with cognitive variables, neuroticism, mood and psychological distress were estimated in a large population-based cohort (Generation Scotland: Scottish Family Health Study, GS:SFHS). The individuals were divided into those with, and without, depression (*n=*2587 and *n=*16 764, respectively) to test for the interactions between MDD status and schizophrenia risk. Replication was sought in UK Biobank (UKB; *n=*6049 and *n=*27 476 cases and controls, respectively). In both the cohorts, we found significant interactions between SCZ-PRS and MDD status for measures of psychological distress (*β*_GS_=−0.04, *P*_GS_=0.014 and *β*_UKB_=−0.09, *P*_UKB_⩽0.001 for GS:SFHS and UKB, respectively) and neuroticism (*β*_GS_=−0.04, *P*_GS_=0.002 and *β*_UKB_=−0.06, *P*_UKB_=0.023). In both the cohorts, there was a reduction of case–control differences on a background of higher genetic risk of SCZ. These findings suggest that depression on a background of high genetic risk for SCZ may show attenuated associations with distress and neuroticism. This may represent a causally distinct form of MDD more closely related to SCZ.

## Introduction

Major depressive disorder (MDD) has a lifetime prevalence of approximately 16% and is a heritable and severely disabling psychiatric condition.^1^ It is highly clinically heterogeneous, relying on diagnostic criteria that are fulfilled through the presence of an arbitrary threshold of symptoms that lack empirical validation. Further, individuals passing this threshold can have a very different combination of symptoms. As a likely consequence of causal heterogeneity, the progress in our understanding of depression's underlying risk factors and biological mechanisms has been limited.

MDD is characterized by sustained negative affect, psychological distress and by differences in personality and behavioural traits, such as increased levels of the personality trait neuroticism.^2^ People with MDD also manifest, on average, reduced executive function, attention, processing speed and working memory,^3^ but relatively preserved general cognitive ability.^4^ These behavioural and personality features are partly heritable, enduring features of illness that are often genetically correlated with liability to MDD.^5, 6, 7, 8^ Notably however, these clinical and personality associations are not specific to MDD and also occur in other psychiatric disorders including schizophrenia (SCZ).^9, 10, 11^

Although MDD is a notoriously heterogeneous disorder, evidence is beginning to emerge to suggest potentially distinguishable aetiological sub-categories.^12^ For example, heritability estimates are considerably higher for early-onset cases (⩽25 years) in comparison with later-onset MDD.^13^ Early-onset MDD has also been associated with a more malignant form of the disorder, with increased numbers of episodes, greater symptom severity, increased functional and behavioural impairments, and personality traits such as increased neuroticism.^14, 15^ Genetic studies also suggest there may be differential pathways underlying these sub-categories grouped according to the age of onset^16, 17^ or symptom profiles.^12^

Genetic factors account for ~37% of liability to MDD and depressive symptoms.^18, 19^ Genome-wide association studies have indicated that ~30% of the heritability for MDD is accounted for by an additive polygenic genetic architecture, where risk is conferred by an accumulation of many alleles of small effect.^20^ Molecular genetic approaches also demonstrate shared genetic architectures across the disorders.^21^ For example, two papers have shown genetic correlation between MDD and SCZ using both genome-wide association analysis identifying shared risk loci,^21^ or an approach estimating genetic correlation from sampled single-nucleotide polymorphisms (SNPs).^22^ These findings have been interpreted as showing that there are genetic variants that raise liability to both MDD and SCZ. Recently, however, a second possibility has been proposed—that the genetic correlation between SCZ and MDD is caused not by pleiotropy, but by the misclassification of SCZ cases as MDD. This possibility has recently been tested using a novel method aimed at using genotype data to distinguish pleiotropy from heterogeneity, where early evidence suggested that some cases of SCZ may have been misclassified as MDD, though this did not reach significance in this proof of technique study.^23^ If confirmed, this would imply that some cases of MDD would be a hidden ‘forme fruste' of SCZ, or that some individuals with MDD may be on a trajectory towards SCZ, but have yet to manifest the clinical phenotype.

The effects of polygenic risk for SCZ have also been demonstrated on a number of related behavioural and cognitive phenotypes. It has been consistently demonstrated, for example, that polygenic risk for schizophrenia is associated with decreased cognitive ability in healthy control samples.^24, 25, 26, 27^ However, as most studies have examined these relationships in unaffected controls, it is unclear whether the associations between SCZ risk and behaviour/cognitive ability are ameliorated or magnified by the presence of depression. Clarifying this relationship is important because, if MDD consists of a subtype that is closely related to schizophrenia, then that subtype would be expected to show associations with behaviour and cognitive ability that resembled those of SCZ itself.

In the current paper, we sought to stratify depression using a continuous scale of SCZ polygenic risk score (PRS), hypothesizing that case–control differences would depend on the genetic risk for schizophrenia; in other words, that there would be a significant interaction between SCZ-PRS and MDD status. We made two predictions: (1) based on the premise that deficits in cognition are greater in SCZ than in MDD, we first predicted that the high SCZ-PRS and MDD case–control differences for cognitive variables would be greater compared with those with lower SCZ PRS scores; (2) as neuroticism and psychological distress differ to a greater extent in MDD than SCZ,^28^ we also predicted that SCZ-PRS and MDD status interactions would be found for these variables, but that they would be the reverse direction to cognition (attenuated in those with high SCZ PRS). We sought to test these predictions in a population-based cohort study, the Generation Scotland cohort: The Scottish Family Health Study sample (GS:SFHS).^29, 30^ Replication of any significant findings was sought in UK Biobank (UKB).

## Materials and methods

### Main study participants

#### Generation Scotland: the Scottish Family Health Study

GS:SFHS is a family- and population-based cohort study. The individuals were recruited at random through general medical practices across Scotland. Initial data collection for GS:SFHS took place between February 2006 and March 2011. The complete study protocol has been described in detail elsewhere.^29, 30, 31^ Details of the mental health and related assessments are summarized below. Ethical approval was provided by NHS Tayside Research Ethics Committee (reference 05/S1401/89). Written consent for the use of data was obtained from all the participants.

The full cohort consists of 23 690 individuals who were over 18 years at the time of recruitment and 21 516 of these attended the research clinic. The present study includes 19 351 individuals on whom genome-wide genotype data were available (demographic details presented in Table 1). Pedigree information was available for all the participants, and this has subsequently been validated against estimates of relatedness estimated using genome-wide SNP data on 19 995 individuals.

#### Assessment of MDD in GS:SFHS

MDD was diagnosed using the structured clinical interview for the Diagnostic and Statistical Manual of Mental Disorders (SCID).^32^ A brief questionnaire was administered to the participants to screen for MDD. The participants were asked ‘Have you ever seen anybody for emotional or psychiatric problems?' and ‘Was there ever a time when you, or someone else, thought you should see someone because of the way you were feeling or acting?' If they answered yes to either of these questions (21.7% screened positive), they were asked to complete the SCID.^32^ If they answered no to both of these questions, they were assigned control status. Those who completed the SCID but did not meet the criteria for MDD or other major psychiatric disorder were also defined as controls. The individuals who declined to complete the screening questionnaire or the SCID had their MDD status set to missing. The individuals with a diagnosis of bipolar disorder were removed for the purposes of the current investigation.

#### Cognitive assessment in GS:SFHS

The cognitive abilities were assessed using four tests. Immediate and delayed scores from the recall section of one story of the Wechsler Logical Memory III UK test were summed to provide a measure of verbal declarative memory.^33^ The Wechsler Digit Symbol Coding test was used to measure processing speed.^33^ The verbal ability was assessed using the Mill Hill Vocabulary Scale, junior and senior synonyms.^34^ The executive function was measured using the letter-based phonemic verbal fluency test (letters C, F and L, for 1 min each).^35^ All the test scores were scaled to a mean of zero and standard deviation of one. General cognitive ability (g) scores were obtained by conducting principal component analysis of the tests, and saving the scores on the first unrotated principal component, on which all the tests had substantial loadings.^36^

#### Measures of neuroticism and psychological distress GS:SFHS

Measures of psychological distress were assessed using the 28-item General Health Questionnaire (GHQ-28),^37^ which consists of four subscales designed to assess the following: (1) somatic symptoms, (2) anxiety and insomnia, (3) social dysfunction and (d) ‘severe depression'. A single measure of global affective symptoms was derived from item responses, and the standard Likert scoring totals were used in the current study. The Eysenck Personality Questionnaire-Revised Short Form provided a measure of neuroticism.^38^ For the numbers of individuals completing each measure, see Table 2.

#### Genotyping and polygenic profiling in GS:SFHS

The blood samples were collected using standard operating procedures and stored at the Wellcome Trust Clinical Research Facility Genetics Core, Edinburgh (www.wtcrf.ed.ac.uk) where they were genotyped using the IlluminaHumanOmniExpressExome-8v1.0 BeadChip and Infinium chemistry.^39^ The genotypes were then processed using the IlluminaGenomeStudio Analysis software v2011.1. The details of blood collection and DNA extraction are provided elsewhere.^40^

The PRSs were calculated using the method described previously,^41^ using summary data from the most recent genome-wide association studies from the Psychiatric Genomics Consortium (2014). Briefly, the SNPs were excluded if they had a minor allele frequency <1%, deviated significantly from Hardy–Weinberg equilibrium (*P*<1 × 10^−6^) in the total sample of founder individuals, or had a call rate <99%. Clump-based linkage disequilibrium pruning (*r*^2^=0.2, 300 kb window) was performed to create a SNP set in linkage equilibrium. Before creating PRSs, all strand-ambiguous SNPs were removed from the GS:SFHS data set and the PRS for each individual was calculated using the cumulative sum of the number of each SNP allele multiplied by the log of the odds ratio for SCZ across their whole genome. Five SNP set scores were generated using the *P*-value threshold cut-offs of 1, 0.5, 0.1, 0.05 and 0.01. Our primary analyses concerned those SNPs from the Psychiatric Genomics Consortium data that met a significance level of *P*=0.5 consistent with the previous studies.^41^ The findings for other thresholds are contained within the Supplementary Material.

### Replication sample: UKB

This study includes replication data from the UKB Study (http://www.ukbiobank.ac.uk). UKB received ethical approval from the Research Ethics Committee (REC, reference: 11/NW/0382). UKB is a health research resource that aims to improve the prevention, diagnosis and treatment of a range of illnesses. Through UKB, 502 655 community-dwelling participants were recruited between 2006 and 2010 in the United Kingdom.^42^ They underwent cognitive, neuroticism and physical assessments, provided blood, urine and saliva samples for future analysis, gave detailed information about their backgrounds and lifestyles, and agreed to have their health followed longitudinally. For the present study, genome-wide genotyping data were available on 33 525 individuals. This data set comprised individuals who survived the quality control process, were independent of individuals within GS:SFHS, who were unrelated and who did not meet criteria for another major psychiatric disorder. The individuals were assigned to a narrow definition of MDD including cases of moderate or recurrent MDD, (*n=*6049 and *n=*27 476 cases and controls, respectively), see Supplementary Material for further information.

### Cognitive assessment in UKB

Three cognitive tests were used in the present study to generate measures of general intelligence ‘g'. These tests, which cover the cognitive domains of reaction time, memory and verbal-numerical reasoning were collected at the baseline assessment. In addition, the data from a second assessment, which provided measures of processing speed (similar to the Digit Symbol Coding test, the Symbol Digit Substitution test) were also assessed (further details in Supplementary Material). All the test scores were scaled to a mean of zero and standard deviation of 1. General cognitive ability (g) was estimated using the principal component analysis approach described above.^36^

### Measures of neuroticism and psychological distress in UKB

In UKB, the participants completed the Neuroticism scale of the Eysenck Personality Questionnaire-Revised Short Form (EPQ-R Short Form).^43^ As a measure of psychological distress, UKB participants undertook the Patient Health Questionnaire, which is a multipurpose instrument for screening, diagnosing, monitoring and measuring the severity of depression. As in GS:SFHS, the scores were scaled to a mean of zero and standard deviation of 1.

### Genotyping and polygenic profiling in UKB

The details of the array design, genotyping, quality control and imputation have been described previously.^44^ Quality control included removal of participants based on missingness, relatedness, gender mismatch and non-British ancestry. Polygenic profiles were created for SCZ in all the genotyped participants using PRSice.^45^ PRSice calculates the sum of alleles associated with the phenotype of interest across many genetic loci, weighted by their effect sizes estimated from a genome-wide association study of that phenotype in an independent sample. Before creating the scores, the SNPs with a minor allele frequency <1% were removed and clumping was used to obtain SNPs in linkage equilibrium with an *r*^2^<0.25 within a 200 bp window. Multiple scores were then created for each phenotype containing SNPs selected according to the significance of their association with the phenotype. The genome-wide association studies summary data were used to create polygenic profiles for SCZ in the UKB participants, at thresholds of *P*<0.01, *P*<0.05, *P*<0.1, *P*<0.5 and all SNPs. As in the main GS:SFHS analysis, current results focus on *P*<0.5.

### Statistical analysis

All the analyses were conducted in ASReml-R (www.vsni.co.uk/software/asreml, version 3.0). The associations were examined between PRSs for SCZ and cognition (cognitive factor 'g', and contributing tests), psychological distress (GHQ total) and neuroticism (from the Eysenck personality questionnaire) using mixed linear model association analysis in GS:SFHS. Age, age^2^, sex, four-multidimensional scaling ancestry components and polygenic profile scores were entered as fixed effects. As GS:SFHS is a family-based study, to control for relatedness family structure was fitted as a random effect by creating an inverse relationship matrix using pedigree kinship information. SCZ-PRS and MDD status were entered as main effects in the model and PRS × MDD status effects were examined by including the interaction term. Where significant interactions were found, further tests were conducted in controls and MDD groups separately. Wald's conditional F-test was used to calculate the significance of fixed effects. The *P*-values presented are raw *P*-values uncorrected for multiple testing, results were considered significant if they were reported in both the cohorts (*P*<0.05). The reported *β*-values are standardized values. The proportion of phenotypic variance explained by PRS was calculated by multiplying the profile score by its corresponding regression coefficient and estimating its variance. This value was then divided by the variance of the observed phenotype to yield a coefficient of determination between 0 and 1 and converted into a percentage.^46^

Significant association and interaction effects were also explored within the independent sample UKB where equivalent measures were available. In UKB, the polygenic risk profile scores were examined for their association with observed phenotypes and for group interaction effects in ASReml-R using the same methods as above (details in Supplementary Material), but without the inclusion of a genetic relationship matrix owing to the large data set and unrelated nature of the filtered UKB study population used in the current investigation.

## Results

### Demographic, cognitive, trait-related features of MDD and PRS scores in GS:SFHS

The demographic details are presented in Table 1 for GS:SFHS. The groups defined according to depression status were significantly different in terms of age, sex, for the cognitive measures of logical memory and verbal fluency, with the greatest differences between groups for measures of psychological distress (GHQ) and for neuroticism (Table 1), higher in MDD cases. The MDD cases and controls also differed significantly on the SCZ PRS score, with the MDD individuals scoring higher.

The demographic details of the individuals from UKB included in the current study are presented in Supplementary Table 1. There were significant differences between the groups in terms of age and gender. The groups also differed according to psychological distress and neuroticism, with the MDD cases scoring higher than controls. Differences between the groups did not reach significance for the individual cognitive tests, and the MDD cases and controls differed significantly on the SCZ PRS score, with the MDD individuals scoring higher.

### Interaction between MDD status and polygenic risk for schizophrenia, in GS:SFHS and UKB

**Figure 1:**
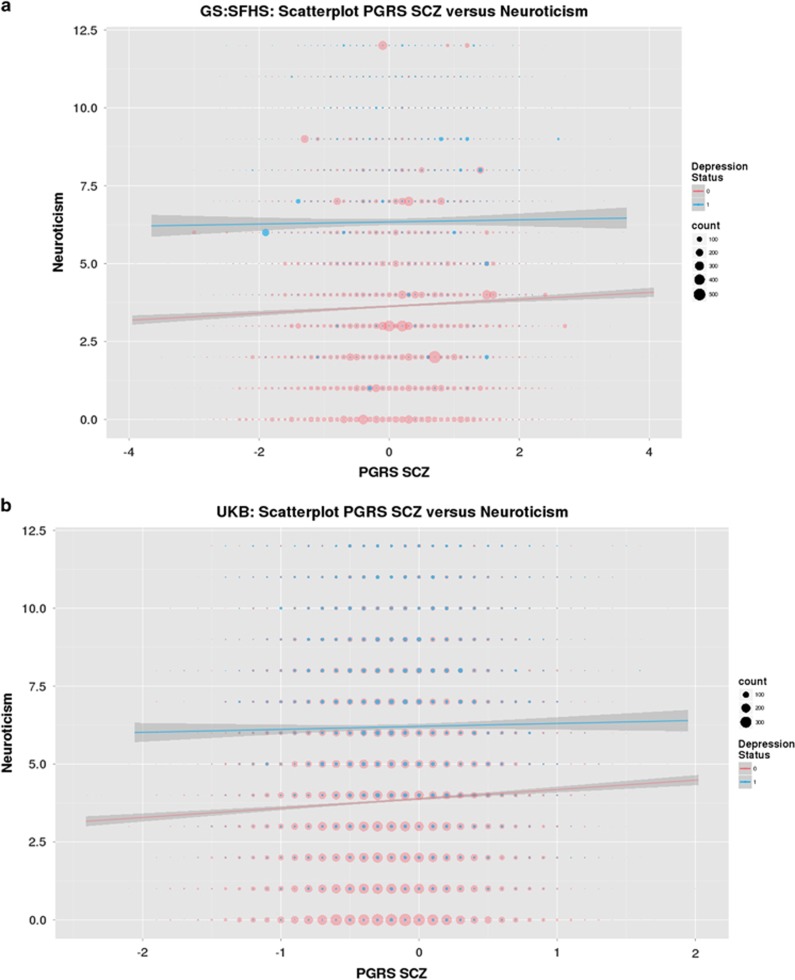
(**a** and **b**) Scatterplot of SCZ PRS and neuroticism according to MDD status in GS:SFHS, and UK Biobank (UKB), respectively. The size of circles represents the degree of overlapping data points. For clarity, histograms of the distribution of neuroticism scores in cases and controls for both cohorts are presented in the Supplementary Figures 1a and b. GS:SFHS, Generation Scotland: Scottish Family Health Study; MDD, major depressive disorder; PRS, polygenic risk score (PGRS in the figure); SCZ, schizophrenia.

The results for tests of interaction are presented in Table 2 and Figures 1a and b. Significant PRS SCZ × group interactions effects were seen across both cohorts for measures of psychological distress (*β*_GS_=−0.0402, *P*_GS_=1.44 × 10^−2^; *β*_UKB_=−0.0903, *P*_UKB_=8.03 × 10^−4^, GS:SFHS and UKB, respectively, Figure 1) and for neuroticism (*β*_GS_=−0.0483, *P*_GS_=1.96 × 10^−3^; *β*_UKB_=−0.0602, *P*_UKB_=2.28 × 10^−2^). A significant interaction was also reported for measures of processing speed in GS:SFHS (*β*_GS_=−0.0313, *P*_GS_=4.31 × 10^−2^), but this was not replicated in the UKB sample.

In terms of the direction of associations within the diagnostic groups, in both GS:SFHS and UKB samples, there was a significant positive association in the control group for measures of psychological distress (*β*_GS_=0.0400, *P*_GS_=1.46 × 10^−8^; *β*_UKB_=0.0827, *P*_UKB_=3.25 × 10^−14^) and for neuroticism (*β*_GS_=0.0369, *P*_GS_=9.15 × 10^−6^; *β*_UKB_=0.0539, *P*_UKB_=4.79 × 10^−19^, GS:SFHS and UKB; see Table 3). There were no significant associations within the MDD groups for either measure, in either cohort (see Table 3, Figures 1 a and b).

**Figure 2:**
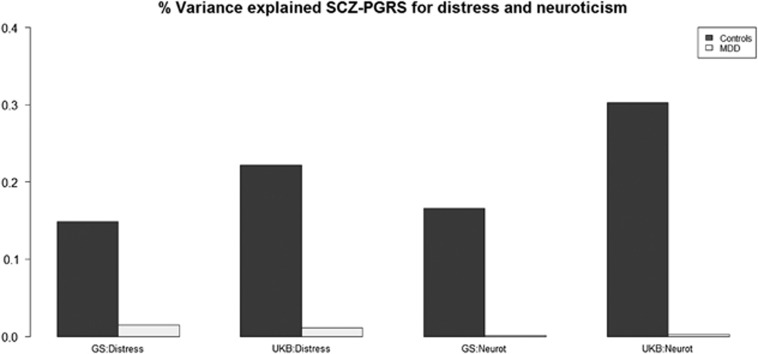
Bar chart of percentage variance explained for distress and neuroticism in GS:SFHS and UK Biobank (UKB). GS:SFHS, Generation Scotland: Scottish Family Health Study; MDD, major depressive disorder; PGRS, polygenic risk score; SCZ, schizophrenia.

In terms of psychological distress, the proportion of variance explained was 0.15 and 0.22% for control individuals, for GS:SFHS and UKB, respectively, and 0.02%, 0.01% for MDD cases. For measures of neuroticism, values were 0.17 and 0.30% in the controls and <0.01% in the MDD cases (see Table 3 and Figure 2).

For both distress and neuroticism, the SCZ-PRS × MDD status interactions remained significant after modelling if the individuals were experiencing a current depressive episode at the time of assessment according to the SCID, and remained significant when modelling if individuals were taking antidepressant medication at the time of assessment according to self-report; full details in Supplementary Material.

## Discussion

Consistent with our predictions, we report MDD case–control differences in neuroticism and distress that were attenuated by higher SCZ PRS scores, found in both the cohorts. We also demonstrate that the greatest variance explained for neuroticism and distress was higher in controls than in the MDD cases, supporting the suggestion that the MDD samples contain a more heterogeneous group comprising differing aetiological subtypes. Although we also report larger MDD case–control differences with respect to cognition in the context of higher SCZ PRS scores in GS:SFHS, this was not replicated in the independent UKB cohort.

### PRS SCZ associations and interactions with clinical and trait features

Neuroticism is considered to be a stable heritable trait^47^ characterized by high tension, irritability, dissatisfaction, shyness, low mood and reduced self-confidence.^38^ Psychological distress is a more generalized measure of psychiatric well-being or psychological health than a specific psychopathological categorization. These two traits have been reported to share strong genetic links,^7^ and there has been considerable support for overlapping genetic risk factors affecting neuroticism and MDD.^48^ Direct links between genetic risk for schizophrenia and these measures, however, are less commonly reported than associations with cognitive deficits, as described above.

These measures demonstrated significant interaction effects between SCZ-PRS and MDD case–control status, seen in both GS:SFHS and UKB cohorts. From Figure 1, it can be seen that for controls, neuroticism scores were positively correlated with SCZ PRSs, which has indeed been previously reported.^49^ However, for MDD cases, the regression line indicated that neuroticism scores did not depend on PRS SCZ. Similar patterns were observed for psychological distress. We are not aware of this relationship having been reported previously. In addition, the variance explained for these measures was greater in control individuals (~0.20%) than in the MDD cases (~0.01%). We suggest that this may be attributable to the aetiologically diverse subgroups within the MDD cases. The origins of these interactions were an attenuation of case–control differences on a background of higher genetic risk of SCZ, seen in both cohorts. Neuroticism and psychological distress may therefore be less closely related to the aetiology of depression in the context of high genetic risk for schizophrenia (see Figure 1). It is, however, also possible that the absence of a similar gradient of effect in the MDD cases could be owing to the substantial effects of depression on distress and neuroticism, leading to a ceiling effect whereby the additional effect of SCZ PRS on these phenotypes is undetectable. However, we consider that this is unlikely as the variance of these measures are not in fact smaller in the MDD groups in comparison with the control group (which would be the case if there were ceiling effects, see Table 1), and so truncation of these scores at the extreme end seems unlikely. Together, these findings suggest that depression on a background of high genetic risk for schizophrenia may represent a somewhat causally distinct form of the condition.

### PRS SCZ associations and interactions with cognition

Previous studies have consistently reported a negative relationship between cognitive function and SCZ PRS, indicating shared genetic risk between SCZ and deficits in cognition.^24, 25, 26, 50^ With the exception of one previous study where genetic loading for SCZ (based on proximal family history) was reported to have a substantial negative impact on neurocognition in mood disorder patients,^51^ we are not aware of any other studies examining this relationship in the context of MDD. In the current study, the data from the four individual subtests that contributed to the general factor indicated a significant interaction between SCZ-PRS and MDD case–control status for the digit symbol coding test. This test is a measure of processing speed, which is known to be significantly impaired in SCZ patients, even in relation to other neuropsychological measures.^52, 53^ Within GS:SFHS, significant negative associations between SCZ-PRS and processing speed were reported in both controls and the MDD cases. In other words, the individuals in GS:SFHS with MDD demonstrated greater deficits in tests of processing speed in those who had higher genetic loading for SCZ versus those at low risk. However, the lack of formal replication means these results should be viewed with caution.

We are aware of one other recent large study using PRSs, including those for SCZ, to determine potential subtypes within MDD.^12^ This study took the approach of determining subgroup classification based on symptom profiles (typical/atypical) and then compared groups using various PRS measures. They reported that typical MDD had a greater genetic overlap with PRS SCZ than atypical MDD, which was more closely related to cardiometabolic PRS such as body mass index and trigycerides.^12^ Although similar, it is however difficult to make direct comparisons between this and the current study, since we used the opposite approach of categorizing according to PRS SCZ and then examined associations with features such as cognition, personality features and psychological distress, which were not described in the Milaneschi *et al.*^12^ study.

### Limitations

Although this is a large and arguably well-powered study with independent replication, there are important limitations that should be considered. Of note is that the data used to derive polygenic scores do not presently fully account for all heritability attributable to common variation, nor do these measures reflect the contribution of rare variation and copy-number variants. In addition, the degree of variance explained by the SCZ PRS score for these phenotypes is relatively modest (0.5–1%). It should be considered, however, that they are consistent with other previously reported figures of 0–2% for similar phenotypes and in similarly unselected population-based cohorts, and that the higher figures reported in the literature (7–18%) generally relate to the phenotype of SCZ case–control status (see ref. 26).

Another limitation relates to consistency of testing between these large data sets. Particularly relevant are the differences in administration of the tests of processing speed. Within GS:SFHS, a pen and paper version of the Digit Symbol Coding task from the Wechsler Adult Intelligence Scale III was used, where participants had a 2 min time limit to complete the task. In UKB, the format of the task was a computerized version where the participant had to click on the number that matched the symbol shown and the participant had 1 min to complete the test. Importantly, there were also fewer individuals included in the DSC assessments in UKB (*n=*7799 controls and *n=*1767 MDD individuals) than for other cognitive measures, and in relation to GS:SFHS (*n=*16 566 controls and *n=*2566 MDD individuals) as this test was conducted at a follow-up assessment rather than at baseline.

The approach we have currently applied consisted of presenting two separate analyses for GS:SFHS and UKB, declaring significance when both the studies reported findings with *P*<0.05. However, we further note that a more stringent approach would be to apply multiple comparison correction within each cohort separately. Applying such a correction for the summary cognitive measure and measures of clinical trait features, the main interactions for distress and neuroticism remained significant in both the cohorts (GS:HFHS, *P*_FDR corrected_=0.0216, *P*_FDR corrected_=0.0059; in UKB *P*_FDR corrected_=0.0024, *P*_FDR corrected_=0.0342, for distress and neuroticism, respectively (FDR, false discovery rate)).

In summary, we consider the main novel finding is not just the significant relationship between PRS SCZ and neuroticism in controls, but the lack of that clear relationship in MDD, inferring heterogeneity. These findings are consistent with a model in which genetic risk for schizophrenia predicts depressive traits in the general population, but that neuroticism and psychological distress may be less closely related to the aetiology of depression on a background of high genetic risk for schizophrenia. This may represent a somewhat causally distinct form of MDD more closely related to SCZ. The study of the genetic basis of variation in such measures is likely to further the understanding of mechanisms by which SCZ genes affect neural function in the context of health and depressive illness.

## Figures and Tables

**Table 1:** Demographics, clinical, behavioural and temperament measures for GS:SFHS individuals in the current study

	*Controls (*n*=16 764)*^a^		*MDD cases (*n*=2587)*^a^		*Significance* T*/Chi^2^ (*P*-value)*
	*Mean*	*s.d.*	*Mean*	*s.d.*	
*Demographics*					
Mean age (years)^a^	47.61	15.27	46.36	12.88	15.73 (7.31 × 10^−5^)
Gender (M:F)^a^	7218:9546	—	746:1841	—	186.54 (2.20 × 10^−16^)
					
*Cognitive*					
Logical memory (*n=*16 764;*n=*2587)	30.70	8.48	31.16	7.89	9.16 (2.48 × 10^−3^)
Digit symbol (*n=*16 566;*n=*2566)	72.32	17.32	71.59	16.21	1.47 (2.25 × 10^−1^)
Mill Hill vocabulary (*n=*16 456;*n=*2563)	30.11	4.72	31.21	4.79^3^	1.70 (1.92 × 10^−^^1^)
Verbal fluency (*n=*16 553;*n=*2575)	39.62	11.67	40.40	11.91	13.53 (2.35 × 10^−4^)
					
*Clinical and trait-related features*					
Psycholog distress (GHQ)	14.93	7.55	22.70^a^	12.62	1797.00 (<1.00 × 10^−100^)
Neuroticism	3.46	2.94	6.46^a^	3.32	2155 (<1.00 × 10^−100^)
					
*PRS measure (measures scaled)*					
SCZ	−0.02	1.00	0.10	1.02	24.00 (9.69 × 10^−7^)

Abbreviations: GHQ, General Health Questionnaire; GS:SFHS, Generation Scotland: Scottish Family Health Study; MDD, major depressive disorder; PRS, polygenic risk score; SCZ, schizophrenia.

a Sample size as indicated unless otherwise specified in descriptive variable column, numbers for controls and MDD, respectively.

Controlled for age, sex, four-multidimensional scaling ancestry components and relatedness.

**Table 2:** PRSs SCZ × MDD status interactions in both samples

	*GS:SFHS* *(Controls* n=*16 764, MDD* n=*2587)*^a^		*UK Biobank* *(Controls* n=*27 476, MDD* n=*6049)*^a^		
	*Statistics (*β*)*	P-*value*		*Statistics (*β*)*	P*-value*
*Cognitive*					
Composite 'g' factor	−0.0220	2.67 × 10^−^^1^	Composite 'g' factor	−0.0434	1.75 × 10^−1^
Logical memory (*n=*16 764; *n=*2587)	−0.0028	8.65 × 10^−1^	Memory	−0.0290	3.54 × 10^−1^
Digit symbol (*n=*16 566; *n=*2566)	**−0.0313**	**4.31** × **10**^−**2**^	Symb digit substitution (*n=*7799; *n=*1767)	0.0197	6.52 × 10^−1^
Mill Hill vocabulary (*n=*16 456; *n=*2563)	0.0009	9.49 × 10^−1^	Reaction time (*n=*27 299; *n=*6008)	0.0119	6.45 × 10^−1^
Verbal fluency (*n=*16 553; *n=*2575)	−0.0010	7.42 × 10^−1^	Verb-num reasoning (*n=*26 847; *n=*5929)	−0.0282	2.92 × 10^−1^
					
*Clinical and trait-related features*					
Psycholog distress (GHQ)	−**0.0402**	**1.44** × **10**^−**2**^	Psycholog distress (PHQ) (*n=*26 581; *n=*5969)	**−0.0903**	**8.03** × **10**^−**4**^
Neuroticism	**−0.0483**	**1.96** × **10**^−**3**^	Neuroticism	**−0.0602**	**2.28** × **10**^−**2**^

Abbreviations: GHQ, General Health Questionnaire; GS:SFHS, Generation Scotland: Scottish Family Health Study; MDD, major depressive disorder; PHQ, Patient Health Questionnaire; PRS, polygenic risk score; SCZ, schizophrenia; symb, symbol; verb-num, verbal-numerical.

a Sample size as indicated unless otherwise specified in descriptive variable column, numbers for controls and MDD, respectively. Controlled for age, sex, population stratification and relatedness.

Significant interactions are highlighted in bold.

**Table 3:** Associations between PRSs for SCZ with cognitive, clinical and trait-related features of MDD in cases/controls separately

*Generation Scotland: SFHS*						
	*Controls (*n=*16 764)*			*MDD cases (*n=*2587)*		
	β	P-*value*	R^*2*^	β	P-*value*	R^*2*^
*Cognitive*						
** **Composite 'g' factor	−0.0773	2.61 × 10^−17^	0.3470	−0.0906	6.50 × 10^−5^	0.6671
** ** Logical memory	−0.0600	2.33 × 10^−13^	0.3119	−0.0764	3.00 × 10^−5^	0.8353
** **Digit symbol	−**0.0654**	**3.27 × 10**^−^**^20^**	**0.3258**	−**0.0906**	**7.35 × 10**^−^**^8^**	**1.0900**
** ** Mill Hill vocabulary	−0.0251	1.36 × 10^−3^	0.0379	−0.0131	5.01 × 10^−1^	0.0173
** ** Verbal fluency	−0.0152	6.70 × 10^−2^	0.0190	−0.0064	7.54 × 10^−1^	0.0110
						
*Clinical and trait-related features*						
Psycholog distress (GHQ)	**0.0400**	**1.46 × 10**^−^**^8^**	**0.1487**	**0.0111**	**6.99 × 10**^−^**^1^**	**0.0149**
Neuroticism	**0.0369**	**91.15 × 10**^−^**^6^**	**0.1661**	−**0.0002**	**9.91 × 10**^−^**^1^**	**0.0009**

*UK Biobank*						
	*Controls (*n=*27 476)*			*MDD cases (*n=*6049)*		
	β*; z ratio*	P-*value*	R^*2*^	β*; z ratio*	P*-value*	R^*2*^
*Cognitive*						
** **Composite 'g' factor	−0.1203	6.99 × 10^−13^	0.3156	−0.1696	4.12 × 10^−9^	0.6162
** ** Memory	0.0657	1.66 × 10^−^^10^	0.1536	0.0490	2.56 × 10^−2^	0.0863
** ** Symbol digit substitution	−0.1006	2.79 × 10^−^^7^	0.2816	−0.0785	5.00 × 10^−2^	0.1802
** ** Reaction time	0.0390	4.79 × 10^−4^	0.0422	0.0509	3.68 × 10^−2^	0.0692
** **Verbal-numerical reasoning	−0.1101	2.16 × 10^−21^	0.3467	−0.1390	2.88 × 10^−8^	0.5495
						
*Clinical and trait-related features*						
PHQ	**0.0827**	**3.25 × 10**^−^**^14^**	**0.2218**	−**0.0259**	**4.20 × 10**^−^**^1^**	**0.0111**
Neuroticism	**0.0539**	**4.79 × 10**^−^**^19^**	**0.3026**	**0.0055**	**6.82 × 10**^−^**^1^**	**0.0029**

Abbreviations: GHQ, General Health Questionnaire; MDD, major depressive disorder; PHQ, Patient Health Questionnaire; PRS, polygenic risk score; SCZ, schizophrenia; SFHS, Scottish Family Health Study.

*R*^*2*^ represents estimate of variance in trait explained by polygene score in percentage.

Significant interactions are highlighted in bold.
