# Supplementary material for: Dissection of major depressive disorder using polygenic risk scores for schizophrenia in two independent cohorts
Source: Transl Psychiatry. 2016 Nov 1;6(11):e938–. doi: 10.1038/tp.2016.207 (PMC5314119; doi:10.1038/tp.2016.207)

**Supplementary Material**

**Replication Sample: UKBiobank Participants**

This study includes data from the UK Biobank Study (<http://www.ukbiobank.ac.uk>). UK Biobank received ethical approval from the Research Ethics Committee (REC). The REC reference for UK Biobank is 11/NW/0382. UK Biobank is a health resource for researchers that aims to improve the prevention, diagnosis, and treatment of a range of illnesses. UK Biobank participants were identified through NHS patient registers and were eligible to participate if they were aged 40-69 and living within a reasonable travel distance to an assessment centre. Around 5 million participants were mailed an invitation letter and approximately 502,655 were recruited (UK Biobank 2011a). They underwent cognitive and physical assessments, provided blood, urine, and saliva samples for future analysis, gave detailed information about their backgrounds and lifestyles, and agreed to have their health followed longitudinally.

**Current Sample:** In the current study participants were excluded as cases and controls if they had ever had a diagnosis from health records of bipolar disorder, multiple personality disorder, schizophrenia, autism, intellectual disability, or Parkinson’s disease; if they had ever had a prescription for antipsychotic or mood stabilising medication; if they self-reported bipolar disorder, schizophrenia, or Parkinson’s disease; or if a touchscreen questionnaire assessment indicated bipolar disorder (Smith et al 2013). Additionally, participants were excluded as controls if they had ever had a diagnosis of an anxiety disorder, a mood disorder, or major depressive disorder; if they had ever been prescribed antidepressant or anxiolytic medication; or if they self-reported depression.

**Sample overlap:** The UK Biobank sample was filtered for internal relatedness and for overlap with the Generation Scotland sample. An optimal subset of unrelated individuals was constructed within the UKB sample using GCTA. Without linking identifiers across study samples, we calculated the genetic relatedness between UK Biobank and Generation Scotland participants. We excluded any UK Biobank participant who had relatedness > 0.0442 with any Generation Scotland participant.

**MDD definition:** The MDD phenotype was based on the putative MDD definition established in Smith et al (2013) using responses to a touchscreen questionnaire (UK Biobank 2011b), from self-report information, and from inpatient records via linkage to hospital episode data. For the analysis, participants with putative recurrent depression (moderate or severe) were classified as cases. Participants with no indications of depression or who had only a single reported episode were classified as controls. Exclusions were made on the basis of self-report information and linked health records

**Cognitive phenotypes**

Four cognitive tests were used in the present study. These tests, which cover four important cognitive domains, were Reaction Time (n= 496,891; of whom 111 484 also had genotyping data), Memory (n= 498,486; 112,067 with genotyping), and Verbal-numerical Reasoning (n= 180,919; 36,035 with genotyping) collected at baseline assessment, and the Symbol Digit Substitution test (n=123,502; 37,399 with genotyping) collected at follow-up assessment. The Reaction Time test was a computerized ‘Snap’ game with eight experimental trials, in which participants were to press a button as quickly as possible when two ‘cards’ on screen were matching. In the Memory test, participants were shown a set of twelve cards (six pairs) on a computer screen for five seconds, and had to recall which were matching after the cards had been obscured. We used the number of errors in this task as the (inverse) measure of Memory ability. The Verbal-numerical Reasoning task involved a series of thirteen items assessing verbal and arithmetical deduction. Finally, the Symbol Digit Substitution test is a test of processing speed based on using codes to link symbols to numbers. We used the number of symbol digits matches made correctly. This was the number of times a participant correctly matched a symbol with a digit summed across all the rounds of the test that they performed, excluding the initial 8 items corresponding to training.

**Genotyping and quality control**

152 729 UK Biobank blood samples were genotyped using either the UK BiLEVE array (N = 49 979)22 or the UK Biobank axiom array (N = 102 750). Details of the array design, genotyping, quality control and imputation are available in a publication22 and in the Supplementary Materials. Quality control was performed by Affymetrix, the Wellcome Trust Centre for Human Genetics as previously (cite Saskias paper); this included removal of participants based on missingness, relatedness, gender mismatch, non-British ancestry, and other criteria, see (Hangenaars et al)

Associations were examined between PRSs for SCZ and BD and baseline measures of cognition (cognitive factor 'g', and contributing tests), and on measures of neuroticism (from the Eysenck personality questionnaire) using mixed linear model association analysis. Age, age^2, sex, ten multidimensional scaling ancestry components and polygenic profile scores were entered as fixed effects. Wald’s conditional F-test was used to calculate the significance of fixed effects.

**Results modelling current depression and medication**

SCZ-PRS MDD case-control interactions remained significant when modelling both current depressive episode, as reported in the SCID, and when modelling current ant-depression use. For psychological distress: β=-0.0822, p=4.21x10^-2^, β=-0.0317, p=4.98x10^-2^ for current depression and antidepressant medication respectively. For neuroticism: β=-0.0865, p=6.20x10^-2^, β =-0.0415, p=5.15 x10^-3^ for current depression and antidepressant medication respectively.

**References**

Smith, D. J., Nicholl, B. I., Cullen, B., Martin, D., Ul-Haq, Z., Evans, J., et al. (2013). Prevalence and Characteristics of Probable Major Depression and Bipolar Disorder within UK Biobank: Cross-Sectional Study of 172,751 Participants. PLoS ONE, 8(11), e75362. DOI:10.1371/journal.pone.0075362

UK Biobank (2011a) Protocol for a large-scale prospective epidemiological resource. Available: http://www.ukbiobank.ac.uk/wp-content/uploads/2011/11/UK-Biobank-Protocol.pdf

UK Biobank (2011b) Touchscreen questionnaire. Available: http://www.ukbiobank.ac.uk/wp-content/uploads/2011/06/Touch_screen_questionnaire.pdf

**Supplementary** **Table 1**: Demographics for UK Biobank

|  | **Controls** (n=27,476)* | | | | **MDD cases** (n=6049)* | | | | | **Significance**  T / Chi 2 (p value) | |  |
| --- | --- | --- | --- | --- | --- | --- | --- | --- | --- | --- | --- | --- |
|  | Mean | | St dev | | Mean | | St dev | | |  | |  |
| **Demographics** | | | | | | | |  | | |  | |
| Mean age (yrs)* | | 57.50 | | (7.96) | | 55.86 | | (7.84) | 200.43 (2.20x10^-16^) | | | |
| Gender (M:F)* | | 13925:13551 | | - | | 2276:3773 | | - | 337.81(2.20x10^-16^) | | | |
| **Clinical and trait-related features** | | | | | | | |  | | |  | |
| Reaction Time (n=27,299;n=6008) | | 562.02 | | (116.96) | | 564.30 | | (118.76) | 2.07(1.50x10^-1^) | | | |
| Memory | | 0.44 | | (1.04) | | 0.46 | | (1.03) | 0.67 (4.14x10^-1^) | | | |
| Verb-Num Reason (n=26,847;n=5929) | | 6.19 | | (2.11) | | 6.19 | | (2.11) | 0.02 (8.94x10^-1^) | | | |
| Symbol digit substitution (n=7799;n=1767) | | 19.78 | | (5.18) | | 19.79 | | (5.04) | 0.97 (9.76x10^-1^) | | | |
| **Clinical and trait-related features** | | | | | | | |  | | |  | |
| PHQ (health qu) (n=26,581;n=5969) | | 1.39 | | (1.89) | | 2.73 | | (2.65) | 2130.79 (2.20x10^-16^) | | | |
| Neuroticism | | 3.82 | | (3.14) | | 6.19 | | (3.27) | 2986.36 (2.20x10^-16^) | | | |
| **PRS measure** (measures scaled) | | | | | | | |  | | |  | |
| **SCZ** | | -0.22 | | (0.52) | | -0.16 | | (0.52) | 69.84 (2.20 x10^-16^) | | | |

* Sample size as indicated unless otherwise specified in descriptive variable column. Controlled for age, sex, C1-4 and relatedness.

**Supplementary Table 2** Associations between PRSs for SCZ with cognitive, clinical and trait-related features of MDD in both samples.

|  | **GS:SFHS** (n=19,351) | | |  |  | | | | **UK Biobank** (n=33,525) | | | | | | | | | |  |  |  |
| --- | --- | --- | --- | --- | --- | --- | --- | --- | --- | --- | --- | --- | --- | --- | --- | --- | --- | --- | --- | --- | --- |
|  | Beta; z ratio | | p value | R^2^ |  | | | | Beta; z ratio | | p value | | | | | | | | R^2^ |  |  |
| **Cognitive** | | | | | | | |  | | | | | |  | | | |  |  |  |  |
| **Composite 'g' factor** | | **-0.0768**  **-9.4024** | **(6.13x10^-19^)** | **0.3582** | **Composite 'g' factor** | | **-0.1407**  **-9.7709** | | | | | **(1.67x10^-22^)** | | | | | **1.4848** | | |  |  |
| **- Logical memory** | | **-0.0603**  **-8.9719** | **(2.22x10^-17^)** | **0.3202** | **-Memory** | | **-0.0535**  **-9.9008** | | | | | **(4.22x10^-23^)** | | | | | **0.2865** | | |  | |
| **- Digit symbol** | | **-0.0656**  **-11.4732** | **(3.71x10^-25^)** | **0.4020** | **- Symb digit substitution** | | **-0.0869**  **-8.1420** | | | | | **(4.05x10^-16^)** | | | | | **0.7544** | | |  | |
| - Mill Hill vocabulary | | -0.0251  -4.0923 | (1.08x10^-4^) | 0.0627 | -Reaction Time | | 0.0520  9.7161 | | | | | (2.62x10^-22^) | | | | | 0.2700 | | |  | |
| - Verbal fluency | | -0.0072  -1.0822 | (3.06x10^-1^) | 0.0055 | -Verb-Num Reasoning | | -0.1188  -12.1784 | | | | | (4.69x10^-34^) | | | | | 1.4019 | | |  | |
| **Clinical and trait-related features** | | | | | |  | | | |  | | | | |  |  |  |  |  |  |  |
| **Psycholog distress (GHQ)** | | **0.0377**  **5.3485** | **(4.22x10^-7^)** | **0.1474** | **Psycholog distress (PHQ)** | | **0.0739**  **12.4789** | | | | | | **(1.03x10^-35^)** | | | **0.5400** | | | |  |  |
| **Neuroticism** | | **0.0352**  **5.1159** | **(6.02x10^-7^)** | **0.1268** | **Neuroticism** | | **0.0374**  **12.1759** | | | | | | **(4.40x10^-34^)** | | | **0.1398** | | | |  |  |

Results highlighted in bold are consistent across samples*.* R^2^ *=* estimate of variance in trait explained by polygene score in %.

**Supplementary Table 3** Associations between PRS SCZ (weighted threshold method, 4 additional standard thresholds) and cognitive, clinical and trait-related features of MDD in Generation Scotland

|  | **Whole sample** (n=18,954) | | | | **Controls** (n=16,325) | | | | | **MDD cases** (n=2544) | | | | **Interaction** | |
| --- | --- | --- | --- | --- | --- | --- | --- | --- | --- | --- | --- | --- | --- | --- | --- |
|  | Statistics  (Beta  z ratio) | | p value | | Statistics (Beta  z ratio) | | p value | | | Statistics (Beta  z ratio) | p value | | | F value p value | |
| **PRS SCZ threshold S1:p=0.01** | | | | | | | |  | | | | | | |  |
| **Cognitive** | | | | | | | |  | | | | | | |  |
| Composite 'g' factor | | -0.05591  -6.5466 | | (5.96x10^-10^) | | -0.0595  -5.4067 | | (6.52x10^-8^) | -0.07193  -2.547 | | | (1.09x10^-2^) | 1.42 (0.22) | |  |
| - Logical memory | | -0.0580  -8.3177 | | (3.72x10^-15^) | | -0.0623  -7.3250 | | (2.52x10^-13^) | -0.0548  -2.8044 | | | (5.08x10^-3^) | <0.01 (0.84) | |  |
| - Digit symbol | | -0.05250  -8.8420 | | (6.34x10^-17^) | | -0.0526  -7.1161 | | (1.16x10^-12^) | -0.0687  -3.8307 | | | (1.31x10^-4^) | 0.50 (0/48) | |  |
| - Mill Hill vocabulary | | -0.0169  -2.6611 | | (1.84x10^-2^) | | -0.0142  -1.7447 | | (8.11x10^-2)^ | -0.0237  -1.1531 | | | (2/49x10^-1^) | 1.91 (0.17) | |  |
| - Verbal fluency | | -0.0057  -0.8329 | | (4.31x10-1) | | -0.0136  -1.5670 | | (1.71x10^--1)^ | -0.0074  -0.3440 | | | (7.31x10^-1^) | 0.08 (0.78) | |  |
| **Clinical and trait-related features** | | | | | | | |  | | | | | | |  |
| Psycholog distress (GHQ) | | 0.0416  5.6904 | | (7.38x10^-8^) | | 0.0388  5.2980 | | (1.19x10^-7^) | 0.0099  0.3217 | | | (7.48x10^-1^) | 3.69 (0.05) | |  |
| Neuroticism | | 0.0374  5.2415 | | (3.28x10^-7^) | | 0.0373  4.7159 | | (2.43x10^-6^) | -0.0088  -0.3946 | | | (6.93x10^-1^) | 10.73 (<0.01) | |  |
| **PRS SCZ threshold S2:p=0.05** | | | | | | | |  | | | | | | |  |
| **Cognitive** | | | | | | | |  | | | | | | |  |
| Composite 'g' factor | | -0.0630  -7.5529 | | (9.16x10^-13^) | | -0.0640  -5.9649 | | (2.50x10^-9^) | -0.0916  -3.3661 | | | (7.74x10^-4^) | 4.09 (0.03) | |  |
| - Logical memory | | -0.0595  -8.7219 | | (1.64x10^-16^) | | -0.0627  -7.5644 | | (4.14x10^-14^) | -0.0712  -3.7690 | | | (1.68x10^-4^) | 0.13 (0.72) | |  |
| - Digit symbol | | -0.0617  -10.64 | | (8.48x10^-24^) | | -0.0582  -8.0936 | | (6.25x10^-16^) | -0.0983  -5.6667 | | | (1.62x10^-8^) | 8.67 (<0.01) | |  |
| - Mill Hill vocabulary | | -0.0180  -2.898 | | (6.10x10^-3^) | | -0.0164  -2.0612 | | (3.93x10^-2^) | -0.0226  -1.1273 | | | (2.59x10^-1^) | 1.52 (0.21) | |  |
| - Verbal fluency | | -0.0083  -1.2291 | | (2.45x10^-1^) | | -0.0157  -1.8598 | | (6.29x10^-2)^ | -0.0105  -0.5002 | | | (6.17x10^-1^) | 0.48 (0.49) | |  |
| **Clinical and trait-related features** | | | | | | | |  | | | | | | |  |
| Psycholog distress (GHQ) | | 0.0311  4.3562 | | (3.78x10^-5^) | | 0.0335  4.7016 | | (2.61x10^-6^) | 0.0096  0.3234 | | | (7.46x10^-1^) | 2.58 (0.11) | |  |
| Neuroticism | | 0.03885  5.5756 | | (5.97x10^-8^) | | 0.0392  5.0940 | | (3.55x10^-7^) | 0.0106  0.4632 | | | (6.43x10^-1^) | 5.35 (0.02) | |  |
| **PRS SCZ threshold S3:p=0.1** | | | | | | | |  | | | | | | |  |
| **Cognitive** | | | | | | | |  | | | | | | |  |
| Composite 'g' factor | | -0.0680  -8.2126 | | (8.11x10^-15^) | | -0.0712  -6.6799 | | (2.47x10^-11^) | -0.0801  -3.0146 | | | (2.60x10^-3^) | 2.27 (0.13) | |  |
| - Logical memory | | -0.0563  -9.3154 | | (3.78x10^-15^) | | -0.0585  -7.1014 | | (1.30x10^-12^) | -0.0672  -3.6301 | | | (2.89x10^-4^) | <0.01 (0.98) | |  |
| - Digit symbol | | -0.0673  -11.69 | | (2.25x10^-28^) | | -0.0663  -9.2732 | | (2.05x10^-20^) | -0.0924  -5.4396 | | | (5.86x10^-8^) | 3.17 (0.07) | |  |
| - Mill Hill vocabulary | | -0.0222  -3.5984 | | (6.63x10^-4^) | | -0.0215  -2.7163 | | (6.61x10^-3^) | -0.0166  -0.8458 | | | (3.98x10^-1^) | 0.19 (0.07) | |  |
| - Verbal fluency | | -0.099  -1.4788 | | (1.62x10^-1^) | | -0.0187  -2.2288 | | (2.58x10^-2^) | -0.0063  -0.3050 | | | (7.60x10^-1^) | 0.02 (0.89) | |  |
| **Clinical and trait-related features** | | | | | | | |  | | | | | | |  |
| Psycholog distress (GHQ) | | 0.0348  4.8980 | | (3.61x10^-6^) | | 0.0364  5.1394 | | (2.80x10^-7^) | 0.0060  0.2053 | | | (8.37x10^-1^) | 3.54 (0.06) | |  |
| Neuroticism | | 0.0368  5.3207 | | (2.13x10^-7^) | | 0.0376  4.9151 | | (8.98x10^-7^) | 0.0054  0.2559 | | | (7.98x10^-1^) | 7.16 (<0.01) | |  |
| **PRS SCZ threshold S5:p=1** | | | | | | | |  | | | | | | |  |
| **Cognitive** | | | | | | | |  | | | | | | |  |
| Composite 'g' factor | | -0.0660  -8.030 | | (3.11x10^-14^) | | -0.0696  -6.5771 | | (4.95x10^-11^) | -0.0797  -3.0396 | | | (2.39x10^-3^) | 0.89 (0.34) | |  |
| - Logical memory | | -0.0584  -8.6836 | | (2.22x10^-16^) | | -0.0580  -7.0990 | | (1.32x10^-12^) | -0.0745  -4.0838 | | | (4.57x10^-5^) | 0.01 (0.91) | |  |
| - Digit symbol | | -0.0654  -11.45 | | (2.81x10^-27^) | | -0.0656  -9.2403 | | (2.80x10^-20^) | -0.0913  -5.4547 | | | (5.39x10^-8^) | 3.55 (0.06) | |  |
| - Mill Hill vocabulary | | -0.0247  -4.0281 | | (1.39x10^-4^) | | -0.0250  -3.1927 | | (1.41x10^-3^) | -0.0139  -0.7178 | | | (4.79x10^-1^) | 1.92 (0.09) | |  |
| - Verbal fluency | | -0.0057  -0.8630 | | (4.14x10^-1^) | | -0.1433  -1.7214 | | (8.52x10^-2^) | -0.0056  -0.2738 | | | (7.84x10^-1^) | 0.08 (0.78) | |  |
| **Clinical and trait-related features** | | | | | | | |  | | | | | | |  |
| Psycholog distress (GHQ) | | 0.0369  5.2380 | | (7.27x10^-7^) | | 0.0393  5.5917 | | (2.30x10^-8^) | 0.0138  0.4814 | | | (6.31x10^-1^) | 5.29 (0.02) | |  |
| Neuroticism | | 0.0351  5.1094 | | (6.17x10^-7^) | | 0.0366  4.8216 | | (1.44x10^-6^) | 0.0010  0.0493 | | | (9.61x10^-1^) | 9.21 (<0.01) | |  |

* SPQ: subsample only had individualised dimension measures: 10,956 whole sample, 7192 controls, 1227 MDD cases

**Supplementary Table 5** Correlation matrix (p values)

|  | S1 | S2 | S3 | S4 | S5 | C1 | C2 | C3 | C4 |
| --- | --- | --- | --- | --- | --- | --- | --- | --- | --- |
| S1 | 1.00 (<0.01) | 0.87  (<0.01) | 0.82  (<0.01) | 0.74  (<0.01) | 0.74  (<0.01) | -0.04  (1.00) | 0.15  (1.00) | 0.35  (1.00) | -0.07  (1.00) |
| S2 | 0.87  (<0.01) | 1.00  (<0.01) | 0.95  (<0.01) | 0.88  (<0.01) | 0.87  (<0.01) | -0.05  (1.00) | 0.14  (1.00) | 0.27  (1.00) | -0.05  (1.00) |
| S3 | 0.82  (<0.01) | 0.95  (<0.01) | 1.00  (<0.01) | 0.93  (<0.01) | 0.92  (<0.01) | -0.05  (1.00) | 0.13  (1.00) | 0.24  (1.00) | -0.04  (1.00) |
| S4 | 0.74  (<0.01) | 0.88  (<0.01) | 0.93  (<0.01) | 1.00  (<0.01) | 1.00  (<0.01) | -0.06  (1.00) | 0.13  (1.00) | 0.20  (1.00) | -0.03  (1.00) |
| S5 | 0.74  (<0.01) | 0.87  (<0.01) | 0.92  (<0.01) | 1.00  (<0.01) | 1.00  (<0.01) | -0.06  (1.00) | 0.13  (1.00) | 0.20  (1.00) | -0.03  (1.00) |
| C1 | -0.04  (0.85) | -0.05  (0.80) | -0.05  (0.81) | -0.06  (0.76) | -0.06  (0.76) | 1.00  (<0.01) | -0.05  (1.00) | 0.06  (1.00) | 0.15  (1.00) |
| C2 | 0.15  (0.42) | 0.14  (0.46) | 0.13  (0.48) | 0.13  (0.50 | 0.13  (0.50) | -0.05  (0.81) | 1.00  (<0.01) | 0.22  (1.00) | -0.08  (1.00) |
| C3 | 0.35  (0.06) | 0.27  (0.15) | 0.24  (0.20) | 0.20  (0.29) | 0.20  (0.29) | 0.06  (0.76) | 0.22  (0.23) | 1.00  (<0.01) | 0.00  (<0.01) |
| C4 | -0.07  (0.70) | -0.05  (0.80) | -0.04  (0.85) | -0.03  (0.88) | -0.03  (0.89) | 0.15  (0.42) | -0.08  (0.69) | 0.00  (0.99) | 1.00  (<0.01) |

S1-5 represent PRS SCZ thresholds 0.01, 0.05, 0.1, 0.5, 1. C1-4 represent multidimensional scaling ancestry components

**Supplementary Figures 1a,b.** Demonstrating the distributions of neuroticism scores across cohorts


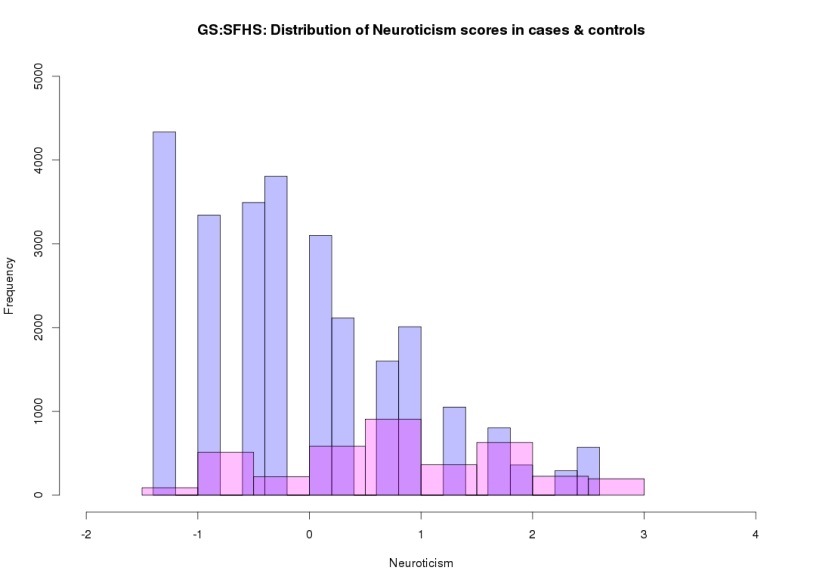

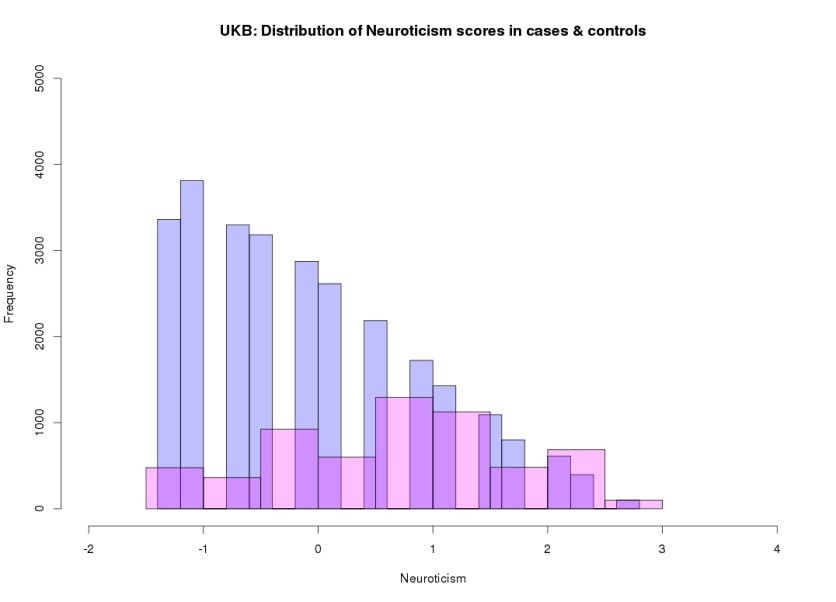

Supplement: Supplementary Information [file tp2016207x1.docx]
